# Rapid identification of a pathogenic variant of PROS1 in a thrombophilic family by whole exome sequencing: A case report

**DOI:** 10.1097/MD.0000000000028436

**Published:** 2021-12-30

**Authors:** Wenwen Zhang, Chen Huang, Wei Zhou

**Affiliations:** a Department of Vascular Surgery, The Second Affiliated Hospital of Nanchang University, Nanchang, Jiangxi, China; b Department of Vascular Surgery, Affiliated Hospital of Nantong University, Nantong, Jiangsu, China.

**Keywords:** *PROS1* gene, rivaroxaban, venous thrombosis, whole exome sequencing

## Abstract

**Rationale:**

Venous thrombosis remains a significant problem in modern days. Genetic factors contribute to a subset of patients with venous thrombosis. It is sometimes challenging to identify the underlying culprit in thrombophilic individuals based on traditional laboratory testing and Sanger sequencing.

**Patient concerns:**

A thrombophilic family presented with multiple venous thrombosis was examined.

**Diagnoses:**

Molecular genetic analysis revealed a pathogenic missense variant of the *PROS1* gene. Based on this finding and clinical manifestations, a final diagnosis of protein S deficiency was made.

**Interventions:**

Whole exome sequencing (WES) of the proband was performed to identify disease-causing variants. Subsequently, Sanger sequencing was performed to validate the variant in the affected members.

**Outcomes:**

Using WES, we rapidly identified a proven pathogenic missense variant (c.1543C > T, p.Arg515Cys) in the sex hormone-binding globulin domain of PROS1, which was confirmed by Sanger sequencing. The decreased level and activity of protein S caused by the variant explained the phenotypes of the family. Patients received rivaroxaban as a long-term anticoagulation therapy and achieved a good prognosis.

**Lessons:**

Our study suggests WES as a rapid search strategy to identify the genetic factors underlying thrombophilic disorders. Patients with venous thrombosis caused by PROS1 mutations could receive rivaroxaban as the first choice of anticoagulation therapy.

## 1 Introduction

Protein S (PS), along with protein C and antithrombin, is one of the 3 main anticoagulant proteins in humans. PS is a vitamin K-dependent glycoprotein that is primarily synthesized in the liver, endothelium, and megakaryocytes. Approximately 60% of PS are non-covalently bound to the C4b binding protein and are not able to inhibit the coagulation process, but the remaining 40% of PS are circulating freely in the plasma and have pivotal anticoagulant roles.^[1]^ PS serves as a cofactor for activated protein C to inactivate factors Va (FVa) and VIIIa (FVIIIa), thus attenuating the activity of tenase and prothrombinase complexes. It can also inhibit thrombin generation by direct interactions with factor Xa (FXa) and FVa in an activated protein C -independent manner.^[2]^ Recently, PS was identified as a cofactor of tissue factor pathway inhibitor, which augments the inhibition of FXa via tissue factor pathway inhibitor.^[3]^

Autosomal dominant inherited PS deficiency (MIM 612336) is a genetic disorder caused by mutations in the structural *PROS1* gene. People with decreased PS expression or activity caused by PS deficiency are at an increased risk of venous thromboembolism (VTE), which primarily includes deep venous thrombosis (DVT) and pulmonary embolism. The prevalence of PS deficiency is estimated to exceed 10% in Asian patients with VTE.^[4]^ PS is encoded by the *PROS1* gene, which is composed of 15 exons and 14 introns spanning over 80 kb of genomic DNA. The most frequent *PROS1* mutation type is missense/nonsense substitutions, followed by splice mutations and small/gross insertions, deletions, or duplications. To date, over 450 genetic mutations have been associated with PS deficiency (HGMD database, http://www.hgmd.org, version released on February 2021).

Patients with venous thrombosis without triggering factors should be included in the genetic analysis of thrombophilia, especially those with a positive family history. Traditionally, we tend to determine the level and activity of coagulation-related proteins, followed by Sanger sequencing of the corresponding gene, in order to identify the underlying culprit. This process is sometimes laborious and time-consuming. With the advent of next-generation sequencing, we can simultaneously examine all related genes and promptly identify pathogenic mutations. In this report, we employed the whole exome sequencing (WES) technique in a thrombophilic family and identified the previously documented pathogenic p.R515C mutation in *PROS1.*

## 2 Case presentation

**Figure 1:**
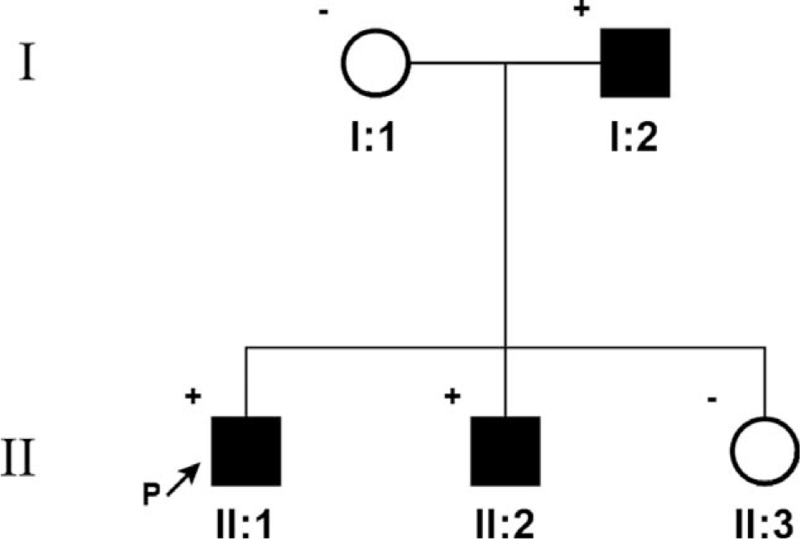
Pedigree; proband is indicated with an arrow. Plus and minus sign indicate presence or absence of the PROS1 mutation, respectively. I:2, upper extremity DVT; II:1, lower extremity DVT; II:2, superior mesenteric venous thrombosis.

The proband was a man of 36 years old who was referred to our center complaining of swelling and pain in the right lower extremity. Ultrasound scanning revealed deep venous thrombosis affecting the femoropopliteal territory. He had no history of surgery, trauma, immobility, or malignancy before the episode. He was treated with low-molecular-weight heparin, followed by oral rivaroxaban. Family history was significant for upper extremity DVT in his father in his early 40s and superior mesenteric venous thrombosis in his brother in his early 30s (Fig. 1). A positive family history and lack of triggering factors regarding venous thrombotic events prompted us to investigate the genetic background. Given the clinical and genetic heterogeneity of thrombophilia disorders, WES was performed. The family's written consent was obtained, and the research protocol was in accordance with our institutional board regulations.

Blood samples were collected from the family members, and genomic DNA was isolated from peripheral blood leukocytes. WES was performed on the proband, as previously described.^[5]^ A total of 200 ng DNA was used for library construction and exons were captured using the SureSelectXT Human All Exon Kit V6 (60 Mb, Agilent Technologies) and sequenced on an Illumina Hiseq 2000 with 2 × 150 bp paired-end reads; sequence reads were demultiplexed, obtaining ∼51 million reads, and quality of the sequencing was evaluated using the FastQC tool (v0.11.5). The raw reads were aligned to the human genome assembly hg19 using BWA enrichment (v0.7.17). Variants were identified using SAMtools (v1.9) and annotated using ANNOVAR. On average, 99.1% of short reads were mapped, reaching an average ∼76-fold depth of coverage. An average of >91% had a base call quality of Q30 or greater.

**Figure 2:**
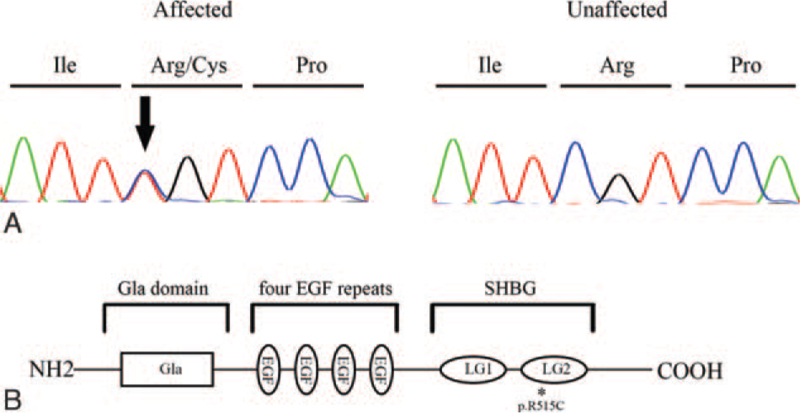
(A) The DNA sequencing chromatograms of the affected and unaffected individuals. The corresponding encoded amino acid is shown above the chromatograms and the arrow denotes the mutated nucleotide. (B) A graphic illustration of the functional domain structure of PROS1 where the mutation p.Arg515Cys is located, as indicated by asterisk. Gla = r-carboxy glutamic acid; LG = Laminin G; SHBG = sex hormone-binding globulin.

Filtering was carried out following strict criteria and consisted of removing any low-confidence variants and excluding variants with a minor allele frequency of ≥0.01% in the 1000 Genomes, ESP, or ExAC databases. Only exonic missense, nonsense, stop-loss, frameshift, and splice variants were considered. We focused on the variants of genes implicated in the blood coagulation pathway. The final analysis revealed a heterozygous missense variant in *PROS1* (c.1543C > T; p.Arg515Cys). This variant was confirmed by Sanger sequencing (Fig. 2A). Sequencing of other family members identified the same variant in his affected father and mother, but not in his unaffected mother and sister, supporting the segregation of the variant with the disease. The variant is located in the C-terminal sex hormone-binding globulin (SHBG)-like domain, which plays a crucial role in protein secretion (Fig. 2B). Moreover, it has been previously described in several reports and included in HGMD. In addition, hematological work-up revealed that PS activity was 16%, free PS level was 36%, and total PS was 42%. Taken together, the p.R515C mutation of PROS1 is responsible for venous thrombosis episodes in the family. He was thereafter administered rivaroxaban after discharge and recovered well during follow-up.

## 3 Discussion

Based on PS level and activity, PSD can be categorized into 3 subtypes: Type I is defined by deficiency of both free and total PS and decreased PS activity; Type II refers to normal free and total PS levels but decreased PS activity; and Type III, decreased free PS levels and decreased PS activity but normal total PS levels. Type I and Type III PS deficiencies are responsible for approximately 95% of PSD and are considered heterogeneous clinical appearances of the same variant.^[6]^ The mutation identified in this study led to decreased free PS levels, total PS levels, and PS activity. Therefore, it is considered to be associated with a type I PS deficiency. The mutation (*PROS1* c.1543C > T, p.Arg515Cys) is located in the C-terminal SHBG-like domain of PS. In vitro experiments revealed intracellular degradation and impaired secretion of mutated PS.^[7]^ Other variants in the SHBG domain have also been shown to affect the secretion of PS, causing severe type I PS deficiency.^[8]^ The above robust evidence verified the pathogenic role of the mutation, which was responsible for the multiple venous thrombotic events in this family.

Hereditary thrombophilia constitutes a major risk factor for unprovoked VTE and its recurrence.^[9]^ Various genetic defects have been identified to account for hereditary thrombophilia, including factor V Leiden, prothrombin G20210A mutation, protein C deficiency, and protein S deficiency.^[10]^ The prevalence of pathogenic genes varies among different ethnic groups. Factor V Leiden and G20210A mutations are almost exclusively identified in Caucasian populations, while deficiency of natural anticoagulants occurs more frequently in Asian populations. However, the mutations observed in this study have been reported in different ethnic cohorts. Borgel et al^[11]^ described an early onset DVT patient in a French family with low PS. Subsequent DNA sequencing revealed a p.R515C mutation. Yamazaki et al^[7]^ identified the same mutation in a Japanese pedigree with hereditary type I PS deficiency. In a screening cohort of German patients with suspected hereditary protein S deficiency, Duebgen et al^[12]^ found the above mutation in patients affected by DVT, pulmonary embolism, and cerebral venous sinus thrombosis. Tang et al^[13]^ also reported the p.R515C mutation in a female DVT patient among Chinese patients with PS deficiency. Interestingly, the mutation was identified in a 12-year-old Japanese girl with infectious endocarditis as the first presentation.^[14]^ In our report, the family members experienced upper- and lower-extremity DVT, along with superior mesenteric venous thrombosis. Taken together, patients harboring the mutation showed venous thrombosis at different locations, regardless of ethnic populations.

Given the high risk of venous thrombosis occurrence and recurrence, accurate diagnosis of PS deficiency represents a crucial part of the effective management of the disease. However, it remains challenging to pinpoint PS deficiency in highly suspected thrombophilic patients, as thrombophilic disorders can be caused by a variety of genes. Traditionally, laboratory testing has always been performed. Once abnormal levels of PS antigen and activity were revealed, subsequent Sanger sequencing was carried out to confirm the diagnosis. Nevertheless, PS levels and activity are often influenced by many factors, such as pregnancy, hormone use, inflammation, anticoagulation therapy, and other acquired conditions, which lead to false-negative and false-positive results.^[15]^ Ultimate diagnosis relies on further genetic screening. The advent of whole-exome sequencing has brought about a paradigm in how medical researchers investigate hereditary disorders. Owing to its high efficacy, WES can promptly identify underlying gene defects. In this report, we utilized WES to rapidly identify the recognized pathogenic mutation of PROS1, which correlated with the phenotypes observed in this family. Identifying mutations can further help guide clinical practice. To date, there is no consensus regarding the anticoagulant choice of hereditary thrombophilia. Several recent reports have demonstrated good clinical outcomes of rivaroxaban in patients with hereditary protein S deficiency.[^16^,^17^] In line with this, our patient received rivaroxaban as a long-term therapy choice and achieved a good prognosis.

## 4 Conclusion

In summary, we report a case of a family with multiple venous thrombotic events. By utilizing WES, we rapidly identified a previously proven pathogenic variant in the SHBG domain of PROS1, which confirmed the diagnosis of hereditary protein S deficiency. Anticoagulation therapy with rivaroxaban resulted in a good prognosis. Identification of the causative genetic defect allows for trigger recognition and gene-based management of thrombophilic patients.

## Author contributions

**Conceptualization:** Chen Huang, Wei Zhou.

**Data curation:** Chen Huang, Wei Zhou.

**Funding acquisition:** Wenwen Zhang.

**Investigation:** Wenwen Zhang, Chen Huang.

**Methodology:** Chen Huang, Wei Zhou.

**Project administration:** Wei Zhou.

**Resources:** Wei Zhou.

**Writing – original draft:** Wenwen Zhang.

**Writing – review & editing:** Wei Zhou.
